# Corpora amylacea in human hippocampal brain tissue are intracellular bodies that exhibit a homogeneous distribution of neo-epitopes

**DOI:** 10.1038/s41598-018-38010-7

**Published:** 2019-02-14

**Authors:** Elisabet Augé, Ingo Bechmann, Núria Llor, Jordi Vilaplana, Martin Krueger, Carme Pelegrí

**Affiliations:** 1 Secció de Fisiologia, Departament de Bioquímica i Fisiologia, Universitat de Barcelona, Barcelona, Spain; 2 Institut de Neurociències, Universitat de Barcelona, Barcelona, Spain; 3 Institute for Anatomy, University of Leipzig, Leipzig, Germany; 4 Secció de Química Terapèutica, Departament de Farmacologia, Toxicologia i Química Terapèutica, Universitat de Barcelona, Barcelona, Spain; 5 Centros de Biomedicina en Red de Enfermedades Neurodegenerativas (CIBERNED), Barcelona, Spain

## Abstract

Corpora amylacea are spherical bodies of unknown origin and function, which accumulate in the human brain during the aging process and neurodegenerative disorders. In recent work, we reported that they contain some neo-epitopes that are recognized by natural IgMs, revealing a possible link between them and the natural immune system. Here, we performed an ultrastructural study complemented with confocal microscopy in order to shed light on the formation of corpora amylacea and to precisely localize the neo-epitopes. We show that immature corpora amylacea are intracellular astrocytic structures formed by profuse cellular debris and membranous blebs entrapped in a scattered mass of randomly oriented short linear fibers. In mature corpora amylacea, the structure becomes compacted and fibrillary material constitutes the principal component. We also determined that the neo-epitopes were uniformly localized throughout the whole structure. All these observations reinforce the idea that corpora amylacea of human brain are equivalent to another type of polyglucosan bodies named PAS granules, present in mouse brain and originated from degenerative processes. All those findings support the hypothesis that corpora amylacea are involved in the entrapment of damaged materials and non-degradable products and have a role in protective or cleaning mechanisms.

## Introduction

Corpora amylacea (CA) are spherical or ovoid polyglucosan bodies (PGBs) that measure 2 to 20 μm in diameter and accumulate primarily in the periventricular and subpial regions of the human brain during the aging process and some neurodegenerative diseases^1,2^. Although they were described by J.E. Purkinje as far back as 1837, their origin and function remain unknown.

Several authors indicate that CA are located within astrocytic processes^3–6^, while others described them as intra-axonal inclusions^7–9^. However, they have not been reported within neuronal perikarya. In any case, it seems that CA are seen less often within neuronal processes than within astrocytic processes^10^. They are positive to periodic acid-Schiff (PAS) staining due to their high polysaccharide content, and when purified by a centrifugation procedure with final extraction in hot water, they yield a clear water-soluble fraction containing 87.9% hexose, 4.7% protein and 2.5% phosphate^2^. As extensively reviewed in Augé *et al*.^11^, the presence of numerous different types of proteins has been described in CA, including (among others) tau protein, amyloid-β peptides, β-tubulin, glial fibrillary acidic protein (GFAP), microtubule-associated protein 2, neuronal nuclei (NeuN) protein, synuclein, S100β, aquaporin 4, ubiquitin, reelin, nestin and some fungal components. As also indicated in Augé *et al*.^11^, the presence of these (and other) components is supported in some studies, but ruled out in others. Most of these components are described using immunohistochemical or immunofluorescence techniques, and in a slightly earlier study we determined that in studies of this kind the positive staining of CA may be produced by contaminant IgMs present in the vial of some primary antibodies^12^. In that study we checked and ruled out (at least at immunohistochemical level) the presence of beta and tau proteins^12^, and in our more recent study we also ruled out the presence of β-tubulin, GFAP, NeuN, S100β and aquaporin 4^11^. On the other hand, we confirmed the presence of ubiquitin, and detected the presence of some components that have not previously been described, such as glycogen synthase and p62^11^.

Ultrastructural analyses revealed that CA are cytoplasmic bodies exhibiting a complex structure in which the most conspicuous component is a compact mass of randomly oriented short linear fibers (8–12 nm). They are regularly rounded, non membrane bound, and are often surrounded by glial fibrils^5^. In an ultrastructural study with biopsy material from the vestibular root entry zone in cases of Ménière’s disease, Sbarbati *et al*.^6^ observed the presence of CA in astroglial processes and suggested that they escape across the glia-limiting lamina into the pial connective tissue or subpial space. As CA accumulate primarily in the periventricular and subpial regions, it appears that this may be a feasible escape route into the cerebrospinal fluid^1^. Accordingly, it has been suggested that CA may be involved in the trapping and sequestration of potentially hazardous products^1^ and they may act as a system that cleans the central nervous system^6,12^.

CA have been associated with PAS granules, degenerative granular structures that appear progressively with age in the mouse brain^12,13^. The analysis of the PAS granule ultrastructure has been subject to extensive studies. PAS granules contain a central core of electron-dense crystalline-like fibrillary deposits that appear to be composed of degenerated membrane-like structures, surrounded by a halo or translucent region that is separated by a slightly discontinuous plasma membrane, thus confirming the intracellular location of these structures^14–16^. The presence of GFAP fibrils in the halo of the granule, the glycogen accumulations and the specific astrocyte-astrocyte junctions with adjacent membranes made it possible to determine that the granules were located in astrocytic processes. Although mature PAS granules are highly structured, immature PAS granules do not present the compact central core, and are characterized by sparse fibrils or scattered membranous-like structures in which some components as those formed from degenerating mitochondria can be integrated. Thus, PAS granules seem to be formed via the aggregations of damaged components of various kinds^15^. Although mouse PAS granules and human CA may be equivalent structures, and so the genesis of CA may be similar to that of PAS granules, their formation has not yet been described. One of the objectives of the present study is to identify CA during their genesis and describe them at ultrastructural level. Since the number of CA in the human brain increases during aging and during the progression of neurodegenerative diseases, we assume that the brain samples from these donors present not only a high number of already constituted CA but also a number of CA in formation.

We recently reported that both CA and PAS granules contain neo-epitopes that can be recognized by natural IgMs^12,17,18^. Neo-epitopes are epitopes that originate *de novo* in certain circumstances and can trigger an immune response. Natural IgM antibodies are antibodies that have been fixed by natural selection during evolution and are thus interspecific; they are present in the blood plasma from birth without prior contact with external antigens. Accordingly, we found that the IgMs directed against the neo-epitopes sited on CA and PAS granules are present in sera from different species such as human, mouse, rat, goat or rabbit, and also in sera from both human umbilical cord and mouse maintained under specific and opportunistic pathogen-free conditions, i.e., before external antigen exposure. Thus, the presence of neo-epitopes that can be recognized by natural IgMs on both CA and PAS granules indicates that organisms are permanently equipped with antibodies prepared to react against them. These studies highlighted a new link between these age-related deposits and the innate immune system. In the case of PAS granules, ultrastructural studies revealed that the neo-epitopes are mainly located in the core of the granules, specifically in the fibrillary or membranous-like fragments^15^. However, the location of the neo-epitopes present on CA has not been described yet.

Therefore, the present study aimed to investigate the ultrastructure of CA in order to shed light on its formation and compare it with that of mouse PAS granules, and to determine the location of neo-epitopes in CA using immunofluorescence and electron microscopy in human autoptic brain tissue.

## Results

### Ultrastructure of *corpora amylacea* from human hippocampal brain

**Figure 1:**
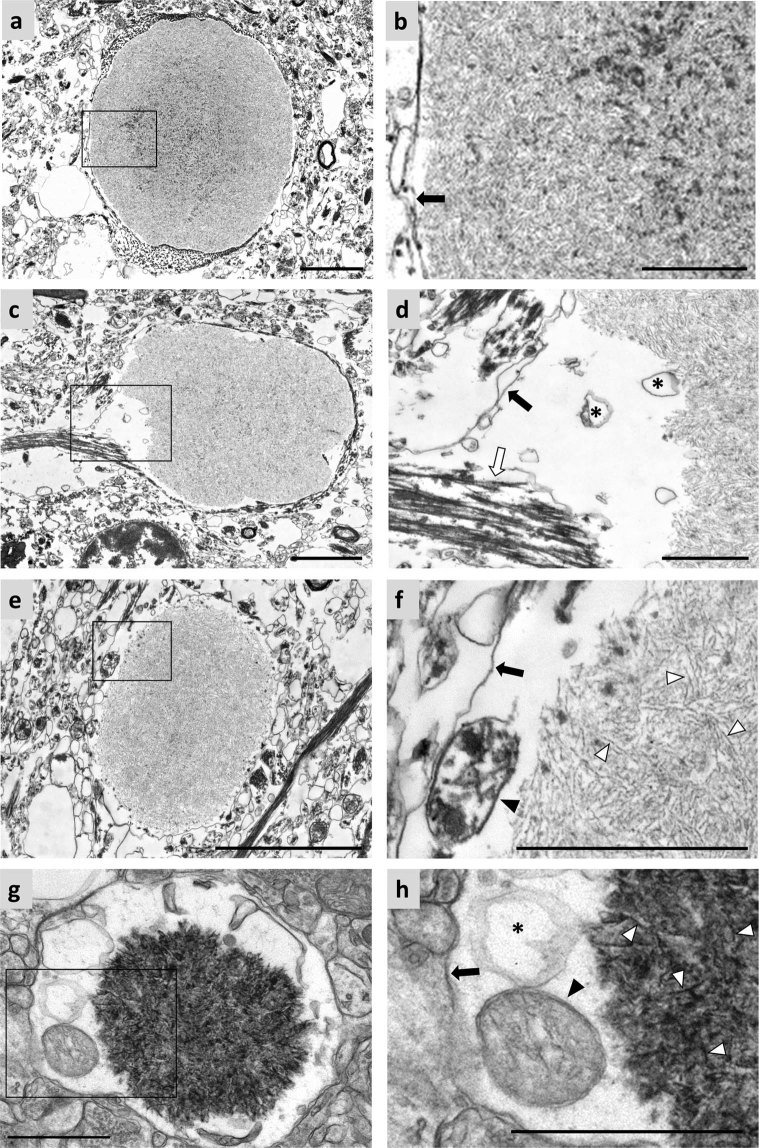
Electron microscopy images of hippocampal CA from human brain and PAS granules from mouse brain. (**a,c** and **e**) Images of different CA. As shown in (**b**) (inset of **a**), CA consist of short linear structures that appear to be concentrated in the central part and which are surrounded by plasma membrane (black arrow). As shown in (**d**) (inset of **c**), membranous structures form irregularly shaped blebs (asterisks), as well as intermediate filaments (white arrow) around CA, which is also surrounded by plasma membrane (black arrow). In (**e**) (inset of **f**), the presence of a plasma membrane (black arrow) surrounding the CA confirms their intracellular location. Some mitochondria (black arrowhead) can be observed near the body. A PAS granule from mice brain can be observed in (**g**). As shown in (**h**) (inset of **g**), the PAS granule is surrounded by plasma membrane (black arrow) and is formed by fibrillary structures (white arrowheads), with membranous blebs (asterisk) and mitochondria (black arrowheads) near it. Differences in the staining intensity between CA and PAS granules are due to methodological procedures (see text), while differences in the quality of the ultrastructure within the surrounding neuropil are related to the unavoidable post mortem delay in human samples. Scale bar in **(a**,**c** and **e)**: 3 µm. Other scale bars: 1 µm. (**g**,**h**) Adapted from AGE 2014, 36:9690, with kind permission of the American Aging Association.

Hippocampal regions from 4% formaldehyde-stored brains of three donors were selected and sectioned to perform native electron microscopy analysis and characterize CA. These structures were observed in the brains of all donors, and they appeared mainly located in the periventricular region of hippocampal region. Except for the cases that will be discussed later, they were all similar to the ones previously described in the literature and which are summarized in Fig. 1. Briefly, they were encircled by a plasma membrane (black arrows in Fig. 1b,d and f), which confirms their intracellular and cytosolic location, but they do not have a membrane of their own. In some cases, intracellular fibers compatible with intermediate filaments can be seen reaching the cytoplasm area containing the CA (white arrow in Fig. 1d). This latter observation was also made by Leel-Ossy^3^, who described these filaments as astrocytic GFAP filaments. Therefore, as the CA are located in the cytoplasm of cells that contain GFAP, it must be concluded that some of them, at least, are astrocytic bodies. It can also be observed that CA are constituted by densely packed, randomly oriented short linear fibers (white arrowheads in Fig. 1f), which in some cases seemed to concentrate more in the central region of CA (Fig. 1a). Moreover, mitochondria were recurrently observed around the body (black arrowhead in Fig. 1f), and irregularly shaped membranous blebs were seen near or in contact with the packed fibers (asterisks in Fig. 1d). All these features have also been previously described in PAS granules in mouse brain, which have been suggested to be analogous to CA in human brain. Images of the ultrastructure of PAS granules are presented in Fig. 1g,h (adapted from Manich *et al*.^15^). The images of mouse PAS granules present some differences with respect to those of human CA, due to the processing of the samples. For example, the ultrastructure of the mouse sample is well preserved because of the absence of post-mortem delay. Moreover, mouse brain samples were treated with potassium ferricyanide while human brain samples were not, and so the carbohydrate components of the former were darker than those of the latter. In any event, it can be shown that, as in the case of CA, PAS granules are constituted by randomly oriented short linear fibers (white arrowheads in Fig. 1h) and are encircled by a plasma membrane (black arrow in Fig. 1h); mitochondria are present around the body (black arrowhead in Fig. 1h), and irregularly shaped membranous blebs are observed near or in contact with the packed fibers (asterisk in Fig. 1h). As described in Manich *et al*.^15^, the PAS granule shown in Fig. 1g is a mature granule.

**Figure 2:**
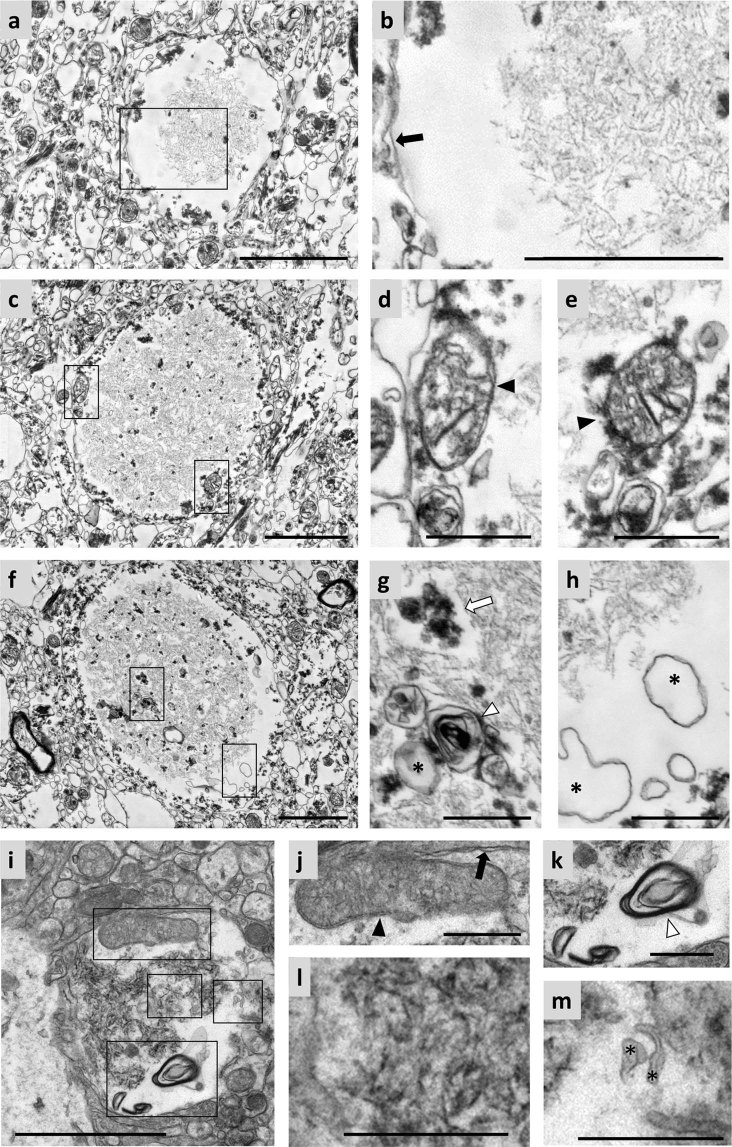
Electron microscopy images of immature CA from human brain and immature PAS granules from mouse brain. (**a**,**c** and **f**) Images of different immature CA. These CA present a different pattern of compaction that could correspond to an early stage in the formation of these structures. This pattern of compaction can be shown magnified in (**b**) (inset of **a**), and the plasma membrane surrounding it is also shown (black arrow). In (**d**,**e**) (insets of **c**), a high number of mitochondria (black arrowheads) can be observed in the space between the CA and the plasma membrane. In (**g**,**h**) (insets of **f**) the presence of some cellular debris (white arrow), multilamellar bodies (white arrowheads) and membranous structures forming blebs (asterisks) are shown. An immature PAS granule from mouse brain can be observed in (**i**). The granule is formed by sparse fibrils (inset **l**), is surrounded by a plasma membrane (black arrow in **j**), and there are mitochondria (black arrowhead in **j**), multilamellar bodies (white arrowhead in **k**) and membranous blebs (asterisks in **m**) around or inside the body. Differences in the staining intensity between CA and PAS granule are due to methodological procedures (see text), while differences in the quality of the ultrastructure within the surrounding neuropil are related to the unavoidable post mortem delay in human samples. Scale bar in (**a**,**c**,**f** and **i**): 2 µm. Other scale bars: 500 nm. (**i**–**m**) Adapted from AGE 2014, 36:9690, with kind permission of the American Aging Association.

On the other hand, a small number of CA, different from the ones described above, have been observed in all brain donors (Fig. 2) and are randomly localized in the periventricular region of hippocampal region. These CA were also intracellular bodies, because of the presence of the plasma membrane around them (black arrow in Fig. 2b) and the mitochondria on the peripheral rim of the body (black arrowheads in Fig. 2d,e). They presented a less compact internal structure and were less well structured than the majority (Fig. 2a,c and f). These CA were formed by sparse fibrils (Fig. 2b), and the irregularly shaped membranous blebs were found not only around the fiber region (asterisks in Fig. 2h) but also inside them (asterisk in Fig. 2g). Inside the fiber region some multilamellar bodies can also be observed (white arrowhead in Fig. 2g), as well as some dark bodies compatible with cellular debris (white arrow in Fig. 2g). While the former type of CA have been compared with mature PAS granules of mouse brain, these rare CA, which are less numerous, less compact and less well structured, are equivalent to the immature ones. An example of an immature PAS granule in mouse brain can be observed in Fig. 2i (also adapted from Manich *et al*.^15^). The PAS granule is inside the cell, as can be deduced from the plasmatic membrane surrounding it (black arrow in Fig. 2j) and the presence of mitochondria (black arrowhead in Fig. 2j); it is formed by sparse fibrils rather than an electron-dense core (Fig. 2l), it also contains irregularly shaped membranous blebs (asterisks in Fig. 2m) and structures associated with degenerative processes like multilamellar bodies (Fig. 2k). All these observations suggest that these CA are in fact immature CA, and that the bodies are formed from the aggregation of certain altered or abnormal components.

In order to extend the description of both types of CA, we also studied the mean diameter of the two populations. We obtained a mean Feret’s diameter of mature CA (n = 31) of 12.13 μm (±0.69 μm), with a maximum diameter of 18.41 μm and a minimum of 4.49 μm, and obtained a mean Feret’s diameter of immature CA (n = 10) of 5.37 μm (±0.77 μm), with a maximum diameter of 11.36 μm and a minimum of 1.87 μm. The Student’s t-test for non-paired data showed statistically significant differences between the Feret’s diameters of the two types of CA, being t_39_ = 5.18 and p < 0.001. We thus concluded that mature CA are larger than immature ones, indicating that during their maturation not only do the fibrillary structures become compacted but also their numbers increase.

### Localization of neo-epitopes in *corpora amylacea*

**Figure 3:**
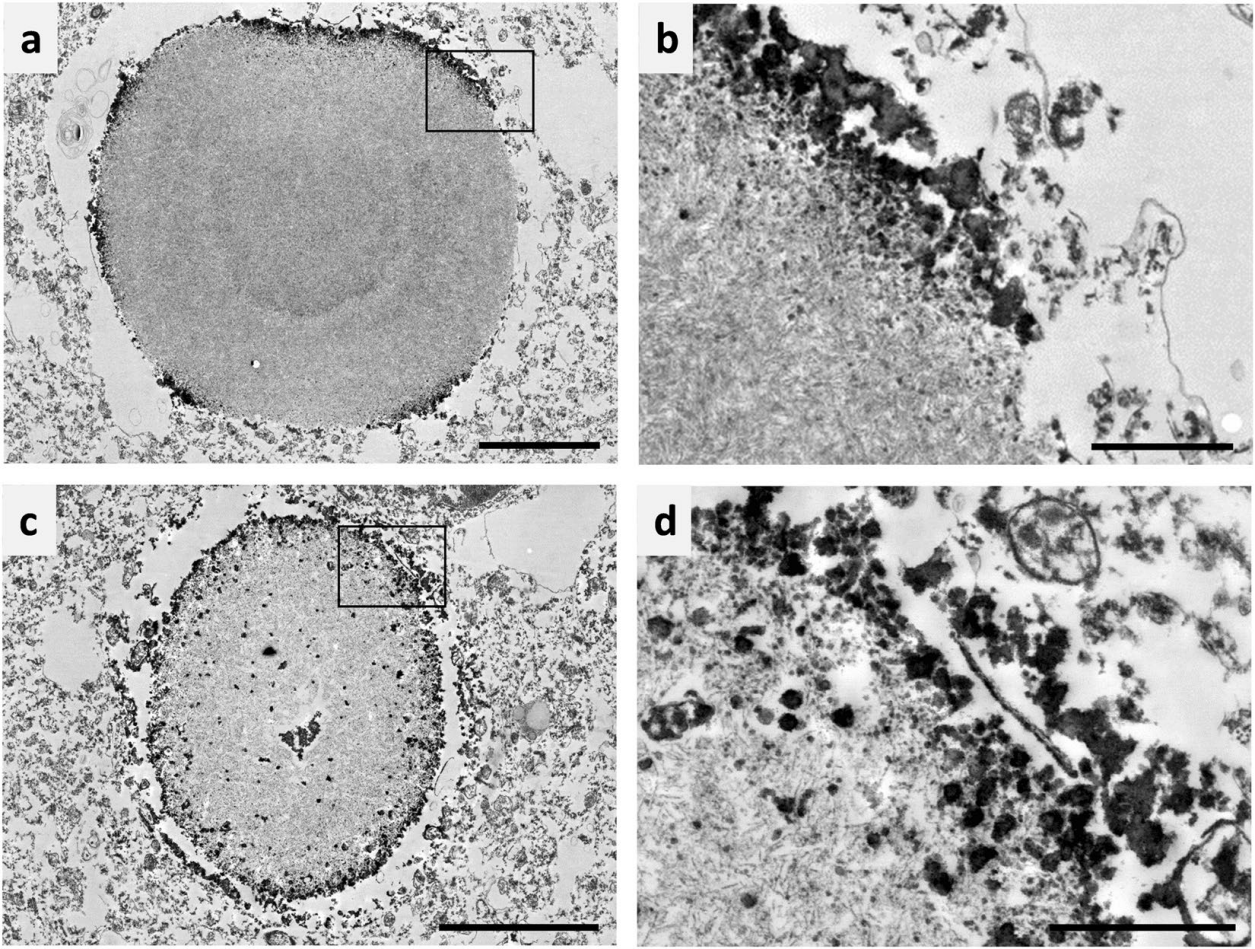
Electron microscopy images of hippocampal corpora amylacea (CA) from human brain immunostained with IgMs. The ultrathin sections were obtained from vibratome sections already stained for IgMs. It is shown that in both the mature (**a**) and the immature (**c**) CA, the IgM staining (shown as dark regions due to DAB grains) was confined mainly to the periphery of the body, while the core of the structure was free from IgM immunoreactivity. (**b**,**d**) are insets of (**a**,**c**), respectively. Scale bar in (**a**,**c**) 5 µm. Scale bar in (**b**,**d**) 1 µm.

Electron-microscopic analysis of ultra-thin sections obtained from vibratome sections immunostained with IgM directed against the neo-epitopes revealed that the IgM staining was confined to the periphery of the CA, while the core of the structures regularly appeared to be devoid of IgM immunoreactivity in both the mature and the immature CA (Fig. 3a,c respectively). The ultrastructure, though, was not completely well preserved, in part due to the staining procedures and to the immunoperoxidase reaction, and the surrounding plasma membrane of CA was often partially lost.

**Figure 4:**
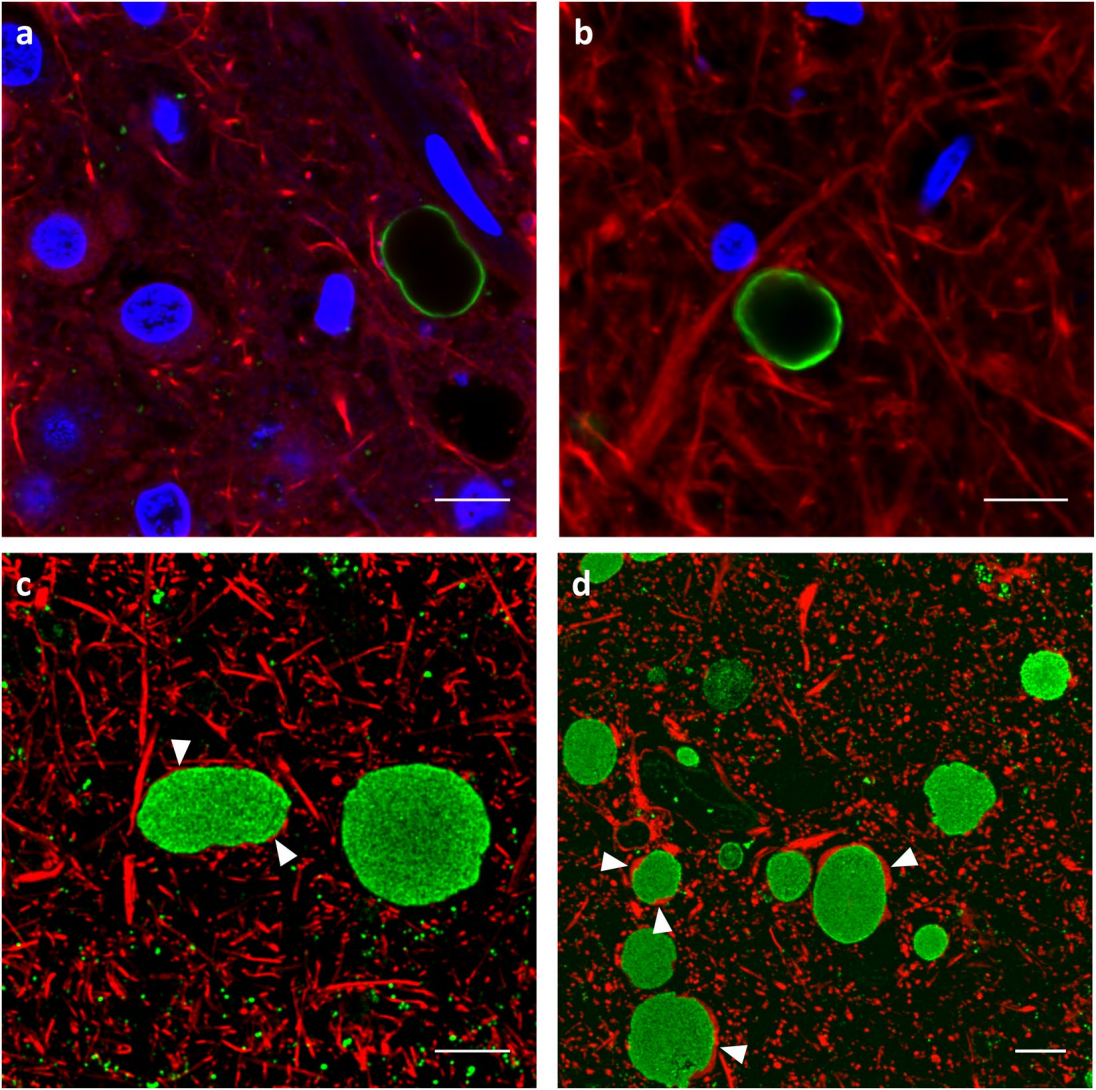
Confocal microscopy images of hippocampal corpora amylacea (CA) from human brain immunostained with IgMs. (**a**,**b**) 60 μm vibratome sections simultaneously immunostained with mouse IgMs against neo-epitopes (green) and with anti-neurofilament heavy (NEFH) (red). DAPI (blue) was used for nuclear staining. Of note, IgM-related immunolabeling is limited to the outer surface. (**c**,**d**) Semi-thin 500 nm sections simultaneously immunostained with mouse IgMs against neo-epitopes (green) and with anti-glial fibrillary acidic protein (GFAP) (red). These sections are not stained with DAPI. In semi-thin sections, but not in vibratome sections, CA become sliced up and IgMs can reach the center of the structure. These results indicate that neo-epitopes are present in the whole CA. CA appear to be ensheathed in astrocytic GFAP-filaments (arrowheads), but not stained with NEFH. Scale bar: 10 µm.

These findings were confirmed by immunofluorescence labeling of anti-neo-epitope IgMs in combination with anti-NEFH in 60 µm vibratome sections: the IgM-related immunoreactivity was confined to the surface of CA, while the core regularly appeared to be devoid of any immunolabeling (Fig. 4a,b). Importantly, NEFH immunoreactivity did not co-localize with IgM immunolabeling of neo epitopes.

However, the rather peripheral staining pattern may not reflect the real distribution of neo-epitopes throughout the entire body of individual CA. In fact, the densely packed structure is likely to prevent complete penetration of the antibody applied, rendering the immunolabeling in the center of the structures impossible. In order to exclude the possible problems of antibody penetration, we further performed immunofluorescence labeling of IgM-related neo-epitopes in combination with anti-GFAP in serially cut semi-thin sections of 500 nm - 1 µm thickness. As CA measure from 2 to 20 µm in diameter, the ones included in the semithin sections were all sectioned in such a way that their central part was exposed and made available to the primary antibody. In this case, the staining covered the whole body of CA, with a homogeneous immunoreactivity that included the core regions, thus indicating that the neo-epitopes are present in the whole CA (Fig. 4c,d). Moreover, and in line with the NEFH-related immunoreactivity, immunolabeling for GFAP did not co-localize with IgM immunoreactivity, although individual CA appeared to be ensheathed in astrocytic GFAP-filaments.

## Discussion

This study was designed to investigate the ultrastructure of CA from human hippocampal brain in order to shed light on its formation and to provide new data about the location of neo-epitopes within these structures.

We show here that most CA consist of a compacted mass of randomly oriented short linear structures, and confirm that they are intracellular formations, as they are surrounded by a plasma membrane which encloses cytoplasmic organelles such as mitochondria. This is consistent with previous descriptions of the ultrastructure of CA^3,5,9^. Further, we observed that the cytoplasmic regions around CA contain intermediate filaments compatible with GFAP filaments at ultrastructural level, and which were shown to represent GFAP filaments at the level of fluorescence microscopy. Thus, although some authors proposed locating CA in axons^7–10^, our results are in line with earlier reports suggesting that the surrounding intermediate filaments correspond to astrocytic GFAP, and support the astrocytic origin of CA^3–5,19^.

Moreover, in this study we observed some CA in early stages. To our knowledge, this is the first study to show the ultrastructure of the immature CA. We observed that immature CA present certain distinctive characteristics with respect to mature CA: early CA contain an inner region that is less structured and less compact than that of mature CA, and also contain mitochondria, cellular debris and membranous blebs located not only surrounding the inner structure but also inside it. Although little is known about the formation of CA and their role, these results are consistent with the suggestions made by other authors indicating that CA are bodies involved in the entrapment of damaged materials and non-degradable products^1,6,12^. In this regard, in a recent study we observed that CA contain ubiquitin and p62, that are proteins both involved in the processing of waste products^11^. Products of oxidative damage from mitochondria, and other potentially damaging materials or non-degradable products generated in the process of aging, are likely to become entrapped in these structures formed in astrocytes and with a content based on polymerized sugar molecules^1,3,12,20^.

A wide range of studies have linked CA to mouse PAS granules^13,21–27^. In previous studies^12,18^, we demonstrated that CA from human brain and PAS granules from mice present neo-epitopes that can be recognized by natural antibodies. The present study identifies other similarities between CA and PAS granules, as indicated by some shared ultrastructural characteristics. Being ensheathed by a plasma membrane, both structures present a fibrillar core, contain membranous structures that form blebs as well as mitochondria adjacent to the core, and are usually surrounded by GFAP filaments. In addition, the ultrastructural study of immature PAS granules demonstrated that such granules presented a central core with sparse fibrils rather than an electron-dense core^15^, in line with the results of the present study with respect to less compact CA. Although size, distribution and some staining properties are different in those two kind of structures and this should be taken into consideration, our results reinforce the idea that CA and PAS granules are similar bodies and that the study of mouse granules might help clarify certain aspects of the nature and development of human CA.

Regarding the neo-epitopes present in CA, immunofluorescence labeling demonstrated that IgM-labeled neo-epitopes are homogeneously distributed throughout the whole body of CA. Although pre-embedding staining in vibratome sections and the subsequent electron microscopy analysis showed that IgM-related immunoreactivity was confined to the periphery of the structure, analysis of semi-thin sections (500 nm to 1 µm) demonstrated that neo-epitopes did not appear exclusively in the periphery of the CA, but also inside the core of the structures. These results are consistent with the study of neo-epitope localization in mouse PAS granules, in which the post-embedding labeling immunoelectron microscopy showed that the neo-epitopes were located mainly in the membrane-like fragments of the whole granule^15^. Although the post-embedding labeling immunoelectron microscopy technique seems to perform better for determining the location of the neo-epitopes, for human brain samples the tissue preservation and manipulation made this procedure difficult, particularly because of the postmortem delay. In fact, few studies of post-embedding labeling immunoelectron microscopy in human brain tissue are available. In any case, the results indicate that neo-epitopes are located throughout the CA and they also show that, in immunohistochemical studies of CA, some epitopes of the central part (compact zone) are not always accessible to the antibodies and can only be labeled when CA has been sliced up. It has to be taken into account, however, that the primary antibodies used for the detection of the neo-epitopes were of IgM type, which have a pentameric or hexameric structure and more than 950 kD. In a recent work, using standard protocols for immunofluorescence and studying the immunolabeling of CA, we observed that IgG antibodies (which are about 150 kD) can penetrate to the CA and stain their internal structure^11^. In that study, we observed that when staining brain sections of 6 µm thick with primary IgG antibodies directed against glycogen synthase (GS), p62 and ubiquitin, the staining of GS and p62 is present only in the periphery of the CA but the staining of ubiquitin is also present in their central part, indicating that IgGs attain this central part. It is also remarkable that in the present work we observe the neo-epitopes (which can be markers of substances to be eliminated by the natural immune system) in the same areas in which ubiquitin (a marker of waste substances) was previously detected.

In conclusion, the present study suggests that CA are intracytoplasmic inclusions presumably located in astrocytes and formed inside the cell, in a process in which some abnormal products become aggregated and some neo-epitopes arise in them, and supports the hypothesis that CA are involved in the entrapment of damaged materials and non-degradable products and can be involved in protective or cleaning mechanisms.

## Methods

### Tissue samples

Human post-mortem brain samples were obtained from three body donors (63–76 years old, 2 males/1 female, without evidence of any neurodegenerative diseases, dementia or neoplasia) from the Institute of Anatomy at Leipzig University after institutional approval for the use of post-mortem tissues from the Institute of Anatomy at Leipzig University and in accordance with the Saxonian Death and Funeral Act of 1994, third section, paragraph 18, item 8. All authors declare that all experiments were conducted according to the principles of the Declaration of Helsinki.

Human brain samples were stored in 4% buffered formaldehyde and tissue was taken from the hippocampal region. Tissue samples were cut at 60 µm on a vibrating microtome (Leica) in phosphate buffered saline (PBS) at pH 7.4.

### Tissue processing for electron microscopy

Some 60 µm vibratome sections were post-fixed for 1 h using 4% paraformaldehyde and 0.1% glutaraldehyde in PBS and incubated thereafter with an anti-tau IgG antibody produced in mouse ascites (clone Tau5, ref. MAB361, lot #2890998, Merck Millipore, dilution 1/100). This antibody was used in a previous work in which it was determined that the vial contained contaminant mouse IgMs directed against the neo-epitopes present in CA^12^, and thus it can be used to label these structures. A goat anti-mouse IgM biotin-conjugated (BA-2020, Vector) was used as secondary antibody and staining was visualized with 3,3′-Diaminobenzidine (DAB). Other vibratome sections were post-fixed for 6 h using a PBS solution with 4% paraformaldehyde and 1% glutaraldehyde. This sections will be referred to as native sections. Some immunostained vibratome sections and native sections were then stained with 0.5% OsO_4_ followed by dehydration in graded alcohol and additional staining with 1% uranyl acetate in 70% alcohol for 60 min. Sections were then embedded in Durcupan (Sigma-Aldrich) between coated microscope slides and cover glasses and the resin was polymerized for 48 h at 56 °C. It was therefore possible to identify and precisely trim areas containing CA at the level of light microscopy. Semi-thin (500 nm and 1 µm) and ultra-thin (55 nm) sections were obtained using an ultra-microtome (Leica Ultracut R, Leica). Ultra-thin serial sections were collected using Formvar-coated single-slot grids and then incubated in lead citrate for 5 min. The ultrastructural analysis was performed with a Zeiss SIGMA electron microscope.

### Tissue processing for immunofluorescence

In order to study the location of neo-epitopes in CA, immunofluorescence studies were performed. Some vibratome sections were blocked and permeabilized with 3% bovine serum albumin (Sigma-Aldrich) in PBS with 0.3% Triton X-100 (Sigma-Aldrich) for 30 min. After washing the sections with PBS, they were incubated with the anti-tau IgG antibody previously referenced, which contains mouse anti-neo-epitope IgMs, and chicken polyclonal 200 kDa neurofilament heavy (NEFH) primary antibody (1/200, OriGene) overnight at 4 °C. Afterwards, the sections were washed and incubated for 2 h at room temperature using fluorochrome-labeled secondary antibodies (Alexa Fluor (AF) 488 goat anti-mouse IgM, 1/250, Jackson ImmunoResearch Laboratories; AF647 goat anti-chicken IgG, 1/250, Invitrogen). 4′6-Diamidin-2-phenylindol (DAPI) was used to visualize nuclei. Omitting the primary antibodies in control experiments resulted in the absence of staining, as expected.

In addition, some semi-thin native sections were used to perform immunofluorescence staining. These sections were obtained as described previously for the electron microscopy study, but OsO_4_ and uranyl acetate staining were omitted and Epon resin was used instead of Durcupan. After removing the resin with a mixture of sodium methoxide solution (Sigma) and a methanol/toluene solution, sections were blocked and permeabilized with 3% bovine serum albumin (Sigma-Aldrich) in PBS with 0.3% Triton X-100 (Sigma-Aldrich) for 30 min. After washing the sections with PBS, they were incubated with anti-tau IgG antibody produced in mouse ascites (1/100, Merck Millipore), which contains mouse anti-neo-epitope IgMs, and rabbit polyclonal α-GFAP primary antibody (1/200, Dako) for 1.5 h at room temperature. Afterwards, the sections were washed and incubated for 1.5 h at room temperature using fluorochrome-labeled secondary antibodies (AF488 goat anti-mouse IgM, 1/250, Jackson ImmunoResearch Laboratories; AF568 goat anti-rabbit IgG, 1/250, Invitrogen).

To assess the fluorescent staining in vibratome and semi-thin sections, confocal laser scanning microscopy (Olympus Fluoview 1000 confocal microscope) and Fluoview software were used.

### Measurement of CA diameter and statistical analysis

The diameters of different CA were measured on electron microscopy images obtained using the Image J program (National Institute of Health, USA). For each CA, we first defined the region of interest (ROI) manually tracing the contour of the CA by using the “freehand selection” tool of the image J program. Thereafter, for each ROI, the Feret’s diameter (the longest distance between any two points along the selection boundary, also known as maximum calliper) was obtained. To compare the Feret’s diameter of mature and immature CA, statistical analyses were performed by means of t-test for independent samples by using Statistica for Windows (Stat Soft Inc.). Differences were considered statistically significant when p < 0.05.
